# ALK-TPM3 rearrangement in adult renal cell carcinoma: a case report and literature review

**DOI:** 10.1186/s13000-019-0879-0

**Published:** 2019-10-18

**Authors:** Jing Yang, Lei Dong, Hong Du, Xiu-bo Li, Yan-xiao Liang, Guo-rong Liu

**Affiliations:** 1 Department of pathology, Guangzhou first people’s hospital, School of Medicine, South China University of Technology, Guangzhou, 510180 Guangdong China; 2 Department of pathology, Ruijin Hospital, Shanghai Jiao Tong University School of Medicine, Shanghai, 200025 China

**Keywords:** Renal cell carcinoma, Anaplastic lymphoma kinase, Cytogenetics, Next generation sequencing

## Abstract

**Background:**

Translocation-associated renal cell carcinoma involving ALK (ALK-tRCC) is a rare subtype of adult renal cell carcinoma (RCC) reported in recent years. It was recognized as a group of emerging /provisional RCC in the latest World Health Organization’s classification (2016).

**Case presentation:**

A new Chinese case of *ALK*-tRCC was reported. The patient was a 58-year-old man with a tumor in kidney. The tumor was composed of sheets of large cells with abundant eosinophilic cytoplasm and indistinct cell borders but conspicuous intracytoplasmic vacuoles. The nuclei were enlarged with a nucleolar of grade 4. Immunohistochemically, tumor cells were diffusely positive for PAX8, keratin (AE1/AE3), epithelial membrane antigen (EMA) and CK7. Fluorescent in situ hybridization (FISH) showed a rearrangement of *ALK* in tumor cells.

**Conclusion:**

*ALK*-tRCC is a rare subtype of adult RCC. Its diagnosis is very difficult because the histological spectrum is very wide. We suggested that RCCs should be screened for ALK expression by immunohistochemistry (IHC) for the patient might benefit from ALK inhibitors therapy.

**Electronic supplementary material:**

The online version of this article (10.1186/s13000-019-0879-0) contains supplementary material, which is available to authorized users.

## Background

Anaplastic lymphoma kinase (*ALK*) translocation was first reported in a distinct subset of anaplastic large cell lymphoma, which is a rare subtype of T-cell lymphoma [1]. Subsequently, *ALK* rearrangements have been found in various types of neoplasm, such as inflammatory myofibroblastic tumor, the diffuse large B-cell lymphoma, plasmacytoma, non-small cell lung carcinoma, esophageal squamous cell carcinoma, breast carcinoma, colonic adenocarcinoma, thyroid carcinoma and, recently RCC [2–10].

Several groups had continuously reported translocation-associated renal cell carcinomas (*ALK*-tRCCs). The first two reported cases were bearing a fusion of *ALK* with *VCL* [9, 10]. They were located in the renal medulla occurring in children affected by sickle-cell trait. Both of them had solid architecture and featured polygonal to spindle-shaped cells with vesicular nuclei and abundant eosinophilic cytoplasm with prominent intracytoplasmic lumina. There was a remarkable lymphoplasmacytic infiltration. Subsequently, 4 cases of RCC associated with *ALK* rearrangements in which *VCL* was not the fusion partner had been reported [11, 12]. All 4 of these neoplasms affected adults without sickle-cell trait, and their morphology lacked common features, except that they all had papillary structures and a variable number of tumor cells with eosinophilic cytoplasm. Therefore, 3 of the neoplasms had been classified as variants of papillary RCC. Afterwards, six more children cases and seven more adult cases were reported. In the children cases, only one of them had a *VCL-ALK* gene fusion with sickle-cell trait, the other five cases had the different fusion gene partners without sickle-cell trait [13–16]. So far, to the best of our knowledge, approximately 19 cases of *ALK*-tRCCs, which had complete clinic and pathologic information, had been reported from U.S.A, Japan, Korean, France, Canada and China (Table 1). Although the number of cases reported had increased in recent years, *ALK*-tRCC still represents a very rare subtype of RCC, especially in adults. So it was recognized as an emerging/provisional subtype of RCC in the latest World Health Organization’s classification [22].

**Table 1:** Clinicopathological and genetic features of ALK-rearranged renal cell carcinoma in 20 cases

No.	Age	Sex	Size (cm)	Furhman Grade	pTNM	Follow-up	Fusion gene	Sickle cell trait
Childern young adults (< 20 years old)								
1 [9]	16	M	6.5	ND	T1N1M0	A (9 m)	VCL	yes
2 [10]	6	M	4.5	ND	T1bN0M0	A (21 m)	VCL	yes
3 [13]	6	M	3	ND	T1aN0M0	A (19 m)	VCL	yes
4 [14]	12	F	6	ND	ND	A (1y)	TPM3	no
5 [15]	16	M	4.5	4	T3aNXMX	ND	TPM3	no
6 [16]	16	F	7	4	T3aN1MX	ND	TPM3	no
7 [15]	14	M	3.7	4	T1aN1MX	ND	TPM3	no
8 [16]	16	M	5.5	3 and 4	T1bN0M0	ND	HOOK1	no
Adults (> 20 years old)								
9 [17]	40	F	4.5	ND	T1bNxM0	A (15 m)	ND	no
10 [18]	55	F	3.1	4	T1aNxM0	A (8 m)	TPM3	no
11 [19]	33	F	ND	3	T2aN1M0	A (27y)	STRN	no
12 [19]	38	M	4.5	4	T1aN2M1	D (3 m)	STRN	no
13 [12]	36	F	4	ND	T1aNxM0	A(2y)	TPM3	no
14 [12]	53	F	2.5	ND	T1aNxM0	A(3y)	EML4	no
15 [20]	49	M	6.4	ND	T1bN1M0	A(24 m)	TPM3	no
16 [20]	52	F	3.5	ND	T1aN0M0	A(8 m)	EML4	no
17 [21]	44	M	3	3	T1aNxM0	A(12y)	ND	no
18 [11]	61	M	5	2	T1bNxM0	D(4y)	ND	no
19 [11]	59	M	5.5	3	T1bNxM0	D(1.4y)	ND	no
20	58	M	2	4	T1aN0M0	A(8 m)	TPM3	no

*ND* Not Done, *A* Alive, *D* Died

We reported herein a new Chinese adult case of RCC with *ALK* rearrangement from Guangzhou, China and a summary of associated clinicopathologic features, biological behavior and molecular genetic changes of *ALK*-tRCC that we have discovered.

## Case presentation

**Fig. 1:**
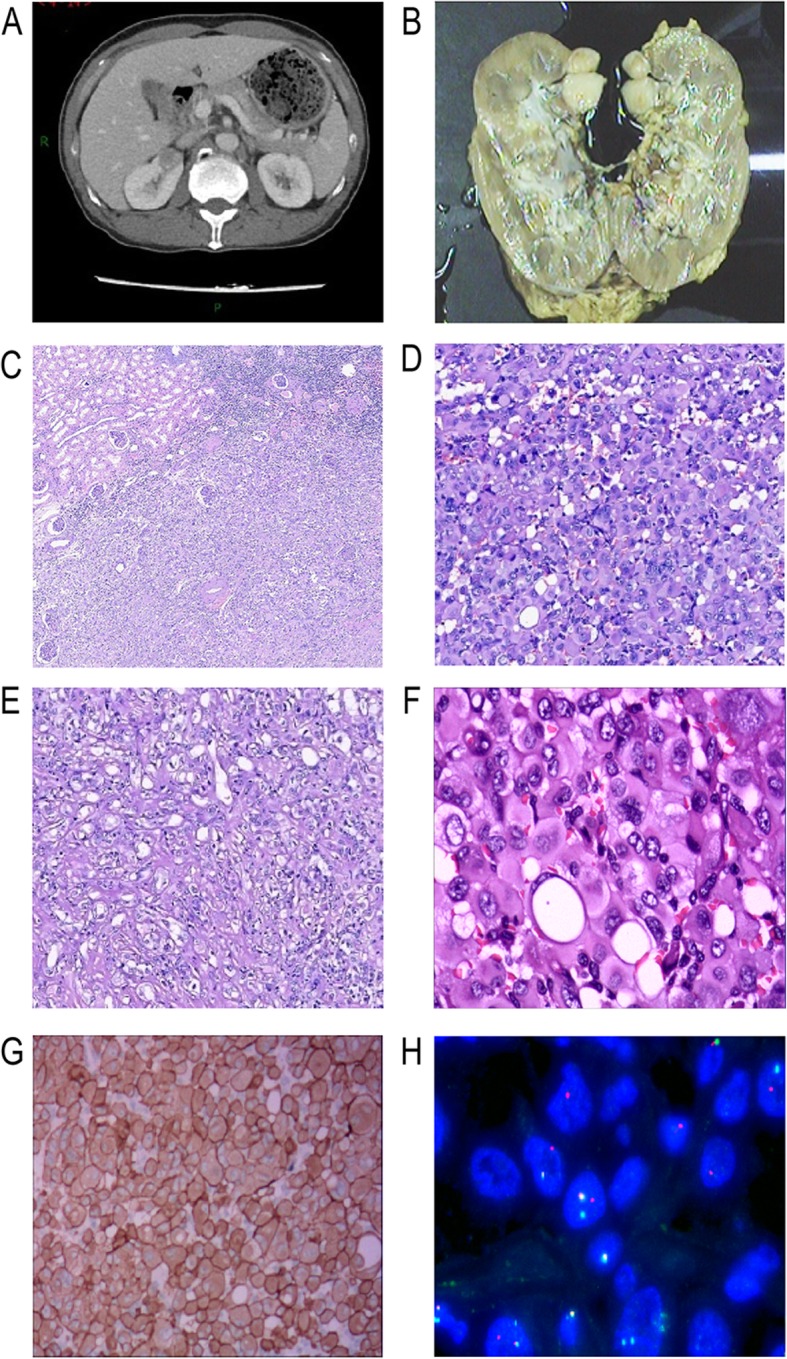
**a** Computed tomography showed a low-density mass (19× 18 × 22 mm) in the upper pole of the right kidney; **b** Grossly, a unique well-circumscribed tumor of 2 cm diameter was under the renal capsule. The cut surface was faint yellow and solid; **c** Histologically, under the ultra low power, the tumor wasclearly demarcated from the surrounding normal renal tissue. (H&E 20×); **d** In the solid growth area, tumor cells were large and polygonal with abundant eosinophilic cytoplasm. Nuclei were round to oval, prominently enlarged and occasionally bizarre, with prominent nucleoli and amounts of clumped to vesicular chromatin (H&E 200×); **e** Histologically, in the tubular growth area, epithelial cells showed smaller in size, less cytoplasm and inconspicuous nucleoli (H&E 200×); **f** Cytoplasmic lumina and nuclear pseudoinclusions were evident. Multinucleated and rhomboid cells were focally noted and corresponded to Fuhrman grade 4 Under the high power, Cytoplasmic lumina and nuclear pseudoinclusions were evident. Multinucleated and rhomboid cells were focally noted and corresponded to Fuhrman grade 4 (H&E 400×); **g** Immunohistochemically, tumor cells showed diffuse and strong positivity for ALK (D5F3), located in the cytoplasm and cell membrane; **h** FISH demonstrated that 53% of the nuclei showed either isolated 3′ *ALK* signals (1R1F, 32%) or break-apart signals (1R1G1F, 21%)

A 58-year-old male patient was found incidentally to have a mass in his kidney during a medical check-up without evidence of sickle cell trait. No distinct clinical symptoms were identified. An abdominal ultrasound revealed an oval hypoechoic mass image (24× 22 mm) with clear-cut margin in the upper pole of the right kidney. Computed tomography confirmed a low-density mass (19× 18 × 22 mm) in the upper pole of the right kidney (Fig. 1a). No metastasis was found. The patient has no familial history of cancer. A right radical laparoscopic nephrectomy was performed. After surgery, he hadn’t taken any ALK inhibitors all along, and he was in good condition 16 months after surgery and showed no evidence of recurrence or metastasis.

Macroscopic examination revealed that there was a 2-cm diameter unique well-circumscribed tumor under the renal capsule. Its cut surface was faint yellow and solid (Fig. 1b). There was no lymph node identified around the perirenal adipose tissue.

Histopathologically, under the ultra-low power, the tumor was clearly demarcated from the surrounding normal renal tissue (Fig. 1c). It was composed of predominantly solid nests, but irregular tubular growth pattern was also admixed. In the solid growth area, tumor cells were large and polygonal with abundant eosinophilic cytoplasm. Nuclei were round to oval, prominently enlarged and occasionally bizarre, with prominent nucleoli and amounts of clumped to vesicular chromatin. Cytoplasmic lumina and nuclear pseudoinclusions were evident (Fig. 1d, f). Multinucleated and rhomboid cells were focally noted and corresponded to Fuhrman grade 4 (Fig. 1f). In the tubular growth area, epithelial cells appeared to be smaller. Less cytoplasm and inconspicuous nucleoli were observed (Fig. 1e). Mitotic figures were scant in two different areas. Thick-walled abnormal blood vessels were obviously seen in the stroma. Numerous lymphocytes, plasma cells and neutrophils were scattered in the stroma. Occasionally, foam cell collections were observed. Desmoplasia was focally present. No mucin deposition and psammoma was observed in the stroma.

Immunohistochemically, tumor cells showed diffuse positivity for PAX8, keratin (AE1/AE3), EMA, CK7, MLH1, PMS2, MSH2 and MSH6, and focal positivity for AMACR and CD10. Staining for SMA, desmin, HMB-45, Melan-A, TFE3, P53, CD34, ERG, CD31, CD117 and S-100 were negative. INI1 was showed diffuse nuclear positivity. Tumor cells also showed diffuse and strong positivity for ALK(Roche, D5F3), which is found to be located in the cytoplasm and cell membrane (Fig. 1g). ALK staining was performed with a BenchMark XT automated staining instrument.

*ALK* rearrangement was confirmed by *ALK* break-apart FISH (Fig. 1h). 53% of analyzed cells showed either isolated 3′ *ALK* signals (1R1F, 32%) or break-apart signals (1R1G1F, 21%). But no rearrangement of the *TFE3* and *TFEB* gene was detected by FISH.

**Fig. 2:**
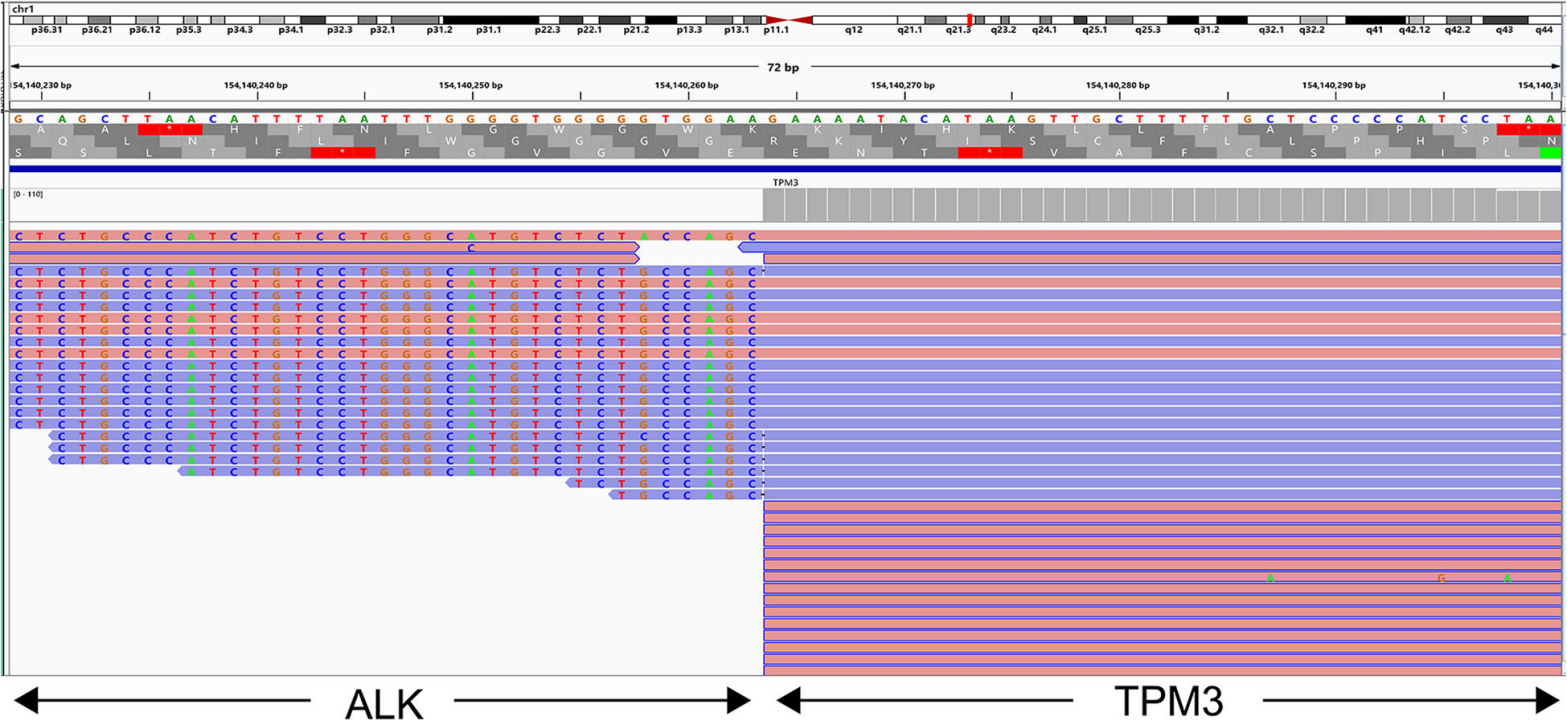
NGS assay detected the fusion of ALK (donor end: chr2: 29447792) and TPM3 (acceptor start: chr1: 154140265) in the tumor visualized in the Integrative Genomics Viewer (IGV, www.broadinstitute.org/igv, human reference genome hg19). The fusion gene showed

Next-generation sequencing was performed with targeted gene capture using ddCAP200 V2 kit(Singlera Genomics, Shanghai Inc., China). This panel covers part or whole exon regions and some intron regions of 216 cancer related genes. A fusion of ALK (donor end: chr2: 29447792) and TPM3 (acceptor start: chr1: 154140265) was detected with frequency 2% (Fig. 2). In addition, 4 genetic variants with uncertain significance were found, including *BARD1* (c.773 T > C), *MSH3* (c.178G > C), *FANCA* (c.1756G > A) and *NF1* (c.4382 T > C) (Additional file 1: Table S1).

## Discussion and conclusions

The dramatic therapeutic benefit of therapies targeting ALK for patients with non-small cell lung carcinoma (NSCLC) driven by ALK fusion is now widely accepted [23, 24]. In recent years, a few of studies indicated that ALK inhibitors, like entrectinib and alectinib, can be dramatically effective for *ALK*-tRCCs [25–27]. Although, the incidence rate of *ALK*-tRCCs is low, the identification and in-depth investigation of *ALK*-tRCCs seems to be very important due to the potentially similar treatment response and prospects. Herein, we summarized the clinicopathologic features, biological behavior and molecular genetic changes of *ALK*-tRCCs.

### Clinical features

By analyzing four large series cohorts of a total 2195 cases being recorded in the past, we found the incidence rate of *ALK*-tRCCs was only about 0.3% (7 out of 2195) in the adults [11, 12, 20, 21]. These cases mainly came from East Asian including Japan, South Korea and China. So far, a total of 12 adult cases had been found, including the case we are reporting here. Another 8 cases occurred in children and adolescents, of which 3 had sickle cell trait and 5 didn’t.

All cases of *ALK*-tRCCs being reported share the commonness of no characteristic clinical symptoms. Painless gross hematuria was the most frequently complaint by the patients. Other symptoms including abdominal discomfort, acute onset varicocele, rash, sweats, weight loss, fatigue, early satiety and anorexia were mentioned occasionally. Or, there were no symptoms at all. They were only found accidently during routine health check-up. Also, there was no preponderance in the gender distribution in the adult cases that the rate of male to female was 1:1. The age range was from 33 to 61 and the mean age was 48. In other 8 children and adolescents’ cases, the age range was from 6 to 16 and the mean age was 13. Three of these cases had sickle cell trait and their ages were 6, 6 and 16 years, respectively. There was no preponderance in the occurrence site that the rate of left kidney and right kidney was 1:1, too (Table 1).

### Pathologic features

Grossly, in most reported ALK-tRCC cases without sickle cell trait, the tumors were well-circumscribed and solid, which were located in the renal cortex. On the other side, in 2 out of 3 cases with sickle cell trait, the tumors were irregular and solid with infiltrative borders located in the renal medulla. The diameter of tumors was from 2 cm to 7 cm and the average diameter was 4.4 cm in these cases without sickle cell trait. In the three cases with sickle cell trait, their diameter was 6.5 cm, 4.5 cm and 3 cm, respectively.

Microscopically, there were consistent characteristic morphologic changes in the three *ALK*-tRCC cases with sickle cell trait, including sheet and solid growth pattern, eosinophilic cytoplasm, intracytoplasmic vacuoles, large vesicular nuclei and abundant lymphoplasmacytic infiltrate. However, *ALK*-tRCC cases without sickle cell trait showed wide spectrum of morphology. Their growth pattern was not only sheet and solid, but also nests, papillary, tubular and cribirform. Nine cases of them had papillary growth pattern. This caused that six *ALK*-tRCC cases were firstly classified as papillary renal cell carcinoma (PRCC). However, papillary structure always mixed with others’ structures. We reviewed and compared the morphological descriptions provided in the 16 previous reports with the present case. We found that most cases shared features of complicated organization structure, abundant eosinophilic cytoplasm, intracytoplasmic lumina, high-grade nuclear features with vesicular chromatin and low mitotic activity. In addition, different levels of lymphoplasmacytic infiltrate, desmoplasia and mucin deposition in and out of tumor cells were noticed in several cases. Large multinucleated tumour cells were often seen. Signet-ring cells and rhabdoid cells were mentioned in the minority of the literatures. So, although *ALK*-tRCCs without sickle cell trait have a wide variety of morphological spectrum, we think these features, especially the wide morphological spectrum and high nuclear grade can give us some diagnostic clues for this tumor.

### Immunohistochemical features

We summed up the immunohistochemical markers of *ALK*-tRCCs without sickle cell trait provided in the 16 previous reports and the present case. We found that the frequency of positive rates of these biomarkers ranked as below: AE1/AE3, vimentin, EMA and INI1 were 100%, ALK was 93.75% (15/16), CK7 was 85.71% (12/14), AMACR was 66.67% (8/12), CD10 was 66.67% (8/12) and TFE3 was 46.15% (6/13). CD117, HMB45 and MelanA were negative in all tested cases. ALK was diffusely and strongly expressed in a cytoplasmic and/or membranous. Therefore, ALK could be the most important marker reminding *ALK*-tRCC. The only lack of *ALK* expression in 16 cases was interpreted as a potential false-negative due to use of an old archival FFPE slide, likely resulting in poor antigen retrieval.

Interestingly, the nuclear expression of TFE3 appeared in 46.15% *ALK*-tRCCs, in absence of TFE3 rearrangement. As everyone knows, the vast majority of TFE3-positive renal tumors are cases of Xp11.2 translocation carcinoma. It could easily become a diagnosis pit without FISH test. AS Paul Scott Thorner et al. had reported that gain of the Xp11 locus has been suggested to be a possible cause of TFE3 expression in absence of *TFE3* structural alteration [15]. Another hypothesis might rely on the presence of a cryptic rearrangement of *TFE3* using FISH technique, such as *RBM10-TFE3* [11]. *NONO-TFE3* fusion may also be difficult to detect [21]. However, it is unlikely that rearrangements of both *ALK* and *TFE3* might be present in the same RCC. So, TFE3 expression may be just constitutional, and dependent upon fixation media/conditions. Different ALK clones were used by different reports, including ALK1, ALK11, 5A4, CD246 and D5F3. OF them, D5F3 is widely acknowledged to be more sensitive than others. So, the true positive rate of ALK-tRCC might be higher than the data reviewed at here. It is no doubt that applying more sensitive antibody and standardized staining instrument would improve the detection rate of ALK-tRCC.

### Molecular genetics features

FISH is the most widely used method to confirm *ALK* gene rupture and rearrangement. *ALK* translocation has been identified in all reported 20 cases by FISH. The fusion partner of *ALK* has been confirmed in 16 out of 20 cases of *ALK*-tRCCs. *VCL,* involved in the first three pediatric cases with sickle cell trait was not found in further cases. The most frequent partner was *TPM3* (8 cases, including our case). The *TPM3-ALK* fusion was previously reported in inflammatory myofibroblastic tumor and anaplastic large cell lymphoma. *TPM3* encodes the tropomyosin 3, an actin-binding protein that plays a role in cytoskeletal microfilament assembly and cell migration [28, 29]. The coiled-coil structure of TPM3 could induce homodimerization of the chimeric TPM3-ALK protein and promote auto-phosphorylation of the ALK catalytic domain [30–33]. An increased metastatic potential has been suggested in cell lines harboring *TPM3-ALK* compared with other *ALK* fusion transcripts [34], and isolated case reports have suggested a more aggressive clinical behavior in ALCL and IMT harboring *TPM3-ALK* fusion transcripts [18, 35, 36]. To date, survival data are available only for five of the 8 *ALK-TPM3*-tRCC patients: they were alive for 8, 12, 24, 24 and 6 months after surgery, respectively (Table 1). Although there was one *ALK-TPM3*-tRCC patient who showed recurrence 1 year after surgery, current data is insufficient for stating a prognostic value correlated to the fusion partner of *ALK* in *ALK*-tRCCs. Other fusion partners of *ALK* included *STRN* (2 cases), *EML4* (2 cases) and *HOOK1* (1 cases). In addition, Ciara Ryan et al. reported one special case demonstrating increased copy number of intact 2p23, the chromosomal region containing the *ALK* gene, rather than exhibiting a chromosomal translocation involving *ALK* [17]*.* Furthermore, Sukov WR et al. also found that *ALK* copy number gain was identified in chromophobe renal cell carcinoma (CCRCC) and PRCC [11]. Therefore, *ALK* expression RCC doesn’t equal to *ALK* translocation RCC.

At the same time, 10 out of 20 cases were tested for *TFE3* breakage by FISH, but all the cases were negative. Our case tested *TFEB* gene breakage too. Similarly, we didn’t find *TFEB* structural alteration. In the case reported by Paul Scott Thorner et al., *TFE3* arrangement wasn’t found, but three and four copies of *TFE3* were observed in 10 and 42% of nuclei. So, Paul Scott Thorner et al. suggested that gain of the Xp11 locus might be a mechanism of *TFE3* positive expression [14].

Karyotypic or genomic data were available in only 5 published cases (Table 1). Four cases showed a balanced [10, 13, 14] or unbalanced [9] translocation. Yohan Bodokh et al. detected chromosomal losses, including loss of chromosome 3, 9, 14 and 21q [19]. The loss of chromosome 3 in RCC is a well characterized feature of clear cell RCC, but it has been occasionally described in non-clear cell RCC, such as papillary RCC, tRCC or unclassified RCC [37–39]. In our case, we detected the *ALK-TPM3* translocation by NGS, in addition some genetic mutations with uncertain significance were found, including *BARD1* (c.773 T > C), *MSH3* (c.178G > C), *FANCA* (c.1756G > A) and *NF1* (c.4382 T > C). The correlation between these genes’ mutation and *ALK*-tRCC occurrence retains unknown. We have performed immunostaining of MisMatch Repair deficiency (dMMR) genes, the results showed all their proteins were expressed in tumor cells. And there was no familial history of neurofibromatosis type 1. So, the current evidences did not support that Lynch Syndrome or NF1 were related with this *ALK*-tRCC.

### Prognosis

Due to the rarity of *ALK*-tRCC, its biological behavior has not been completely elucidated. According to these data (Table 1), the majority of patients (10/14 cases) lived uneventfully without signs of recurrence or metastasis, whether the cases with sickle-cell trail (*n* = 3) or the cases without sickle-cell trail (*n* = 11, which had follow-up data). In addition to 2 initially diagnosed as PRCC cases reported by Sukov et al, they died 4 years and 1.4 years after surgery, respectively. So, Sukov et al proposed that RCC harboring *ALK* rearrangement had a poor outcome [11]. The study of Wenjuan Yu et al suggested the five-year cancer-specific survival rate of *ALK* rearrangement RCC was lower than ISUP G1, G2 and G3 CCRCC and PRCC, but higher than ISUP G4 CCRCC and PRCC based on the report in WHO Classification of Tumor of the Urinary and Male Genital Organs by analyzing reported 10 cases [23]. However, from the limited data, we can’t have a definite result about the prognosis due to the short follow-up time, the small number of cases and the early stage of most tumors. In our case, the patient hasn’t taken any ALK targeting drug till now. He showed no evidence of recurrence or metastasis for 16 months after surgery. It’s worth mentioning that the case reported by Thorner et al. had a recurrence with 2 new lesions 1 year after surgery. And then the patient took an orally available *ALK* inhibitor, which she had been lived for more than 1 year. Afterwards, several studies indicated that ALK inhibitors, like Entrectinib and alectinib, were a well- tolerated therapy with a durable response of patients with advanced *ALK*-tRCCs, compared to standard treatment [25–27]. So, we had reasons to speculate that ALK inhibitors could effectively improve the prognosis of advanced *ALK*-tRCCs.

In conclusion, *ALK*-tRCC is a rare subtype of adult RCC. Its diagnosis is very difficult because the histological spectrum is very wide. However, it is very important and necessary to identify the *ALK*-tRCC because patients with these tumors might benefit from ALK inhibitors therapy. Hence, we suggest that a RCC should be considered to perform ALK immunostaining when it shows high nuclear grade. It will constantly increase the number of *ALK*-tRCC, thus making it possible to profoundly understand this tumor and explore its pathogenesis [40, 41].

## Additional file

Additional file 1: **Table S1.** Genetic mutations with uncertain significance in the reported ALK-TPM3 rearranged renal cell carcinoma. (DOCX 18 kb)

## Data Availability

The datasets used and/or analyzed during the current study are available from the corresponding author on reasonable request.

## Abbreviations

ALK: Anaplastic lymphoma kinase
*ALK*-tRCC: Translocation-associated renal cell carcinoma involving *ALK*
CCRCC: Chromophobe renal cell carcinoma
EMA: Epithelial membrane antigen
FISH: Fluorescent in situ hybridization
IHC: Immunohistochemistry
PRCC: Papillary renal cell carcinoma
RCC: Renal cell carcinoma
RMC: Renal medullary carcinoma

## References

[1] Morris SW, Kirstein MN, Valentine MB, Dittmer KG, Shapiro DN, Saltman DL, Look AT (1994). Fusion of a kinase gene, ALK, to a nucleolar protein gene, NPM, in non-Hodgkin’s lymphoma. Science.

[2] Lawrence B, Perez-Atayde A, Hibbard MK, Rubin BP, Dal Cin P, Pinkus JL, Pinkus GS, Xiao S, Yi ES (2000). TPM3-ALK and TPM4-ALK oncogenes in inflammatory myofibroblastic tumors. Am J Pathol.

[3] Ma Z, Cools J, Marynen P, Cui X, Siebert R, Gesk S, Schlegelberger B, Peeters B, De Wolf-Peeters C (2000). Inv(2)(p23q35) in anaplastic large-cell lymphoma induces constitutive anaplastic lymphoma kinase (ALK) tyrosine kinase activation by fusion to ATIC, an enzyme involved in purine nucleotide biosynthesis. Blood.

[4] Bridge JA, Kanamori M, Ma Z, Pickering D, Hill DA, Lydiatt W, Lui MY, Colleoni GW, Antonescu CR (2001). Fusion of the ALK gene to the clathrin heavy chain gene, CLTC, in inflammatory myofibroblastic tumor. Am J Pathol.

[5] Dirks WG, Fahnrich S, Lis Y, Becker E, MacLeod RA, Drexler HG (2002). Expression and functional analysis of the anaplastic lymphoma kinase (ALK) gene in tumor cell lines. Int J Cancer.

[6] Gascoyne RD, Lamant L, Martin-Subero JI, Lestou VS, Harris NL, Muller-Hermelink HK, Seymour JF, Campbell LJ, Horsman DE (2003). ALK-positive diffuse large B-cell lymphoma is associated with Clathrin-ALK rearrangements: report of 6 cases. Blood.

[7] Soda M, Choi YL, Enomoto M, Takada S, Yamashita Y, Ishikawa S, Fujiwara S, Watanabe H, Kurashina K (2007). Identification of the transforming EML4-ALK fusion gene in non-small-cell lung cancer. Nature.

[8] Lin E, Li L, Guan Y, Soriano R, Rivers CS, Mohan S, Pandita A, Tang J, Modrusan Z (2009). Exon array profiling detects EML4-ALK fusion in breast, colorectal, and non-small cell lung cancers. Mol Cancer Res.

[9] Debelenko LV, Raimondi SC, Daw N, Shivakumar BR, Huang D, Nelson M, Bridge JA (2011). Renal cell carcinoma with novel VCL-ALK fusion: new representative of ALK-associated tumor spectrum. Mod Pathol.

[10] Marino-Enriquez A, Ou WB, Weldon CB, Fletcher JA, Perez-Atayde AR (2011). ALK rearrangement in sickle cell trait-associated renal medullary carcinoma. Genes Chromosom Cancer.

[11] Sukov WR, Hodge JC, Lohse CM, Akre MK, Leibovich BC, Thompson RH, Cheville JC (2012). ALK alterations in adult renal cell carcinoma: frequency, clinicopathologic features and outcome in a large series of consecutively treated patients. Mod Pathol.

[12] Sugawara E, Togashi Y, Kuroda N, Sakata S, Hatano S, Asaka R, Yuasa T, Yonese J, Kitagawa M (2012). Identification of anaplastic lymphoma kinase fusions in renal cancer: large-scale immunohistochemical screening by the intercalated antibody-enhanced polymer method. Cancer.

[13] Smith NE, Deyrup AT, Marino-Enriquez A, Fletcher JA, Bridge JA, Illei PB, Netto GJ, Argani P (2014). VCL-ALK renal cell carcinoma in children with sickle-cell trait: the eighth sickle-cell nephropathy?. Am J Surg Pathol.

[14] Thorner PS, Shago M, Marrano P, Shaikh F, Somers GR (2016). TFE3-positive renal cell carcinomas are not always Xp11 translocation carcinomas: report of a case with a TPM3-ALK translocation. Pathol Res Pract.

[15] Cajaiba MM, Jennings LJ, Rohan SM, Perez-Atayde AR, Marino-Enriquez A, Fletcher JA, Geller JI, Leuer KM, Bridge JA (2016). ALK-rearranged renal cell carcinomas in children. Genes Chromosom Cancer.

[16] Cajaiba MM, Jennings LJ, George D, Perlman EJ (2016). Expanding the spectrum of ALK-rearranged renal cell carcinomas in children: identification of a novel HOOK1-ALK fusion transcript. Genes Chromosom Cancer.

[17] Ryan C, Mayer N, Cunningham J, Hislop G, Pratt N, Fleming S (2014). Increased ALK1 copy number and renal cell carcinoma-a case report. Virchows Arch.

[18] Zhang H, Erickson-Johnson M, Wang X, Bahrami A, Medeiros F, Lonzo ML, Oliveira AM (2010). Malignant high-grade histological transformation of inflammatory myofibroblastic tumour associated with amplification of TPM3-ALK. J Clin Pathol.

[19] Bodokh Y, Ambrosetti D, Kubiniek V, Tibi B, Durand M, Amiel J, Pertuit M, Barlier A, Pedeutour F (2018). ALK-TPM3 rearrangement in adult renal cell carcinoma: report of a new case showing loss of chromosome 3 and literature review. Cancer Genet.

[20] Yu W, Wang Y, Jiang Y, Zhang W, Li Y (2017). Genetic analysis and clinicopathological features of ALK-rearranged renal cell carcinoma in a large series of resected Chinese renal cell carcinoma patients and literature review. Histopathology.

[21] Lee C, Park JW, Suh JH, Nam KH, Moon KC (2013). ALK-positive renal cell carcinoma in a large series of consecutively resected Korean renal cell carcinoma patients. Korean J Pathol.

[22] Moch H, Humphrey PA, Ulbright TM, Reuter VE. WHO classification of tumours of the urinary system and male genital organs. Lyon: International Agency for Research on Cancer; 2016.

[23] Shaw AT, Kim DW, Mehra R (2014). Ceritinib in ALK-rearranged non-small-cell lung cancer. N Engl J Med.

[24] Ou SH, Ahn JS, De Petris L (2016). Alectinib in crizotinib-refractory ALK-rearranged non-small-cell lung cancer: a phase II global study. J Clin Oncol.

[25] Ross JS, Ali S, Fasan O (2017). ALK fusions in a wide variety of tumor types respond to anti-ALK targeted therapy. Oncologist.

[26] Jessica J. Tao, Ge Wei, Roopal Patel et al. ALK fusions in renal cell carcinoma: response to entrectinib. JCO Precision Oncology. 2018;2:1–8. 10.1200/PO.18.00185. 10.1200/PO.18.00185 35135161

[27] Pal SK, Bergerot P, Dizman N (2018). Responses to alectinib in ALK-rearranged papillary renal cellcarcinoma. Eur Urol.

[28] Gunning P (2008). Emerging issues for tropomyosin structure, regulation, function and pathology. Adv Exp Med Biol.

[29] Lees JG, Bach CT, O'Neill GM (2011). Interior decoration: tropomyosin in actin dynamics and cell migration. Cell Adhes Migr.

[30] Wilton SD, Eyre H, Akkari PA, Watkins HC, MacRae C, Laing NG, Callen DC (1995). Assignment of the human a-tropomyosin gene TPM3 to 1q22-->q23 by fluorescence in situ hybridisation. Cytogenet Cell Genet.

[31] Amano Y, Ishikawa R, Sakatani T, Ichinose J, Sunohara M, Watanabe K, Kage H, Nakajima J, Nagase T (2015). Oncogenic TPM3-ALK activation requires dimerization through the coiled-coil structure of TPM3. Biochem Biophys Res Commun.

[32] Coffin CM, Patel A, Perkins S, Elenitoba-Johnson KS, Perlman E, Griffin CA (2001). ALK1 and p80 expression and chromosomal rearrangements involving 2p23 in inflammatory myofibroblastic tumor. Mod Pathol.

[33] Chan JK, Cheuk W, Shimizu M (2001). Anaplastic lymphoma kinase expression in inflammatory pseudotumors. Am J Surg Pathol.

[34] Armstrong F, Lamant L, Hieblot C, Delsol G, Touriol C (2007). TPM3-ALK expression induces changes in cytoskeleton organisation and confers higher metastatic capacities than other ALK fusion proteins. Eur J Cancer.

[35] Shin S, Kim J, Yoon SO, Kim YR, Lee KA (2012). ALK-positive anaplastic large cell lymphoma with TPM3-ALK translocation. Leuk Res.

[36] Hoshino A, Nomura K, Hamashima T, Isobe T, Seki M, Hiwatari M, Yoshida K, Shiraishi Y, Chiba K (2015). Aggressive transformation of anaplastic large cell lymphoma with increased number of ALK-translocated chromosomes. Int J Hematol.

[37] Marsaud A, Dadone B, Ambrosetti D, Baudoin C, Chamorey E, Rouleau E, Lefol C, Roussel JF, Fabas T (2015). Dismantling papillary renal cell carcinoma classification: the heterogeneity of genetic profiles suggests several independent diseases. Genes Chromosom Cancer.

[38] Klatte T, Said JW, Seligson DB, Rao PN, de Martino M, Shuch B, Zomorodian N, Kabbinavar FF, Belldegrun AS (2011). Pathological, immunohistochemical and cytogenetic features of papillary renal cell carcinoma with clear cell features. J Urol.

[39] Malouf GG, Monzon FA, Couturier J, Molinie V, Escudier B, Camparo P, Su X, Yao H, Tamboli P (2013). Genomic heterogeneity of translocation renal cell carcinoma. Clin Cancer Res.

[40] Jeanneau M, Gregoire V, Desplechain C, Escande F, Tica DP, Aubert S, Leroy X (2016). ALK rearrangements-associated renal cell carcinoma (RCC) with unique pathological features in an adult. Pathol Res Pract.

[41] Kusano H, Togashi Y, Akiba J, Moriya F, Baba K, Matsuzaki N, Yuba Y, Shiraishi Y, Kanamaru H (2016). Two cases of renal cell carcinoma harboring a novel STRN-ALK fusion gene. Am J Surg Pathol.

