# Supplementary material for: ALK-TPM3 rearrangement in adult renal cell carcinoma: a case report and literature review
Source: Diagn Pathol. 2019 Oct 18;14:112. doi: 10.1186/s13000-019-0879-0 (PMC6798478; doi:10.1186/s13000-019-0879-0)
Supplement: Supplementary file 1 — Table S1. Genetic mutations with uncertain significance in the reported ALK-TPM3 rearranged renal cell carcinoma. (DOCX 18 kb) [file 13000_2019_879_MOESM1_ESM.docx]

**Additional file 1: TableS1: Genetic mutations with uncertain significance in the reported ALK-TPM3 rearranged renal cell carcinoma:**

|  | **Gene** | **Locus** | **Consequence** | **ExonID** | **Frequency of variant** | **DNA variant** | **Protein variant** | **Accession No.** | **CLIN_SIG** |
| --- | --- | --- | --- | --- | --- | --- | --- | --- | --- |
| **1** | BARD1 | chr2:215645825 | missense_  variant | 4/11 | 0.4968 | ENST00000260947.4:  c.773T>C | ENSP00000260947.4:  p.Ile258Thr | NM_000465.2 | Uncertain significance |
| **2** | FANCA | chr16:89845371 | missense_  variant | 19/43 | 0.4075 | ENST00000389301.3:  c.1756G>A | ENSP00000373952.3:  p.Ala586Thr | NM_000135.2 | Uncertain significance |
| **3** | MSH3 | chr5:79950724 | missense_  variant | 1/24 | 0.508 | ENST00000265081.6:  c.178G>C | ENSP00000265081.6:  p.Ala60Pro | NM_002439.4 | Uncertain significance |
| **4** | NF1 | chr17:29587401 | missense_  variant | 33/57 | 0.4602 | ENST00000356175.3:  c.4382T>C | ENSP00000348498.3:  p.Ile1461Thr | NM_000267.3 | Uncertain significance |
| Notation：Results annoatation software version:Variant Effector Predictor (VEP version 84);Database version: ClinVar (version 201710);Reference genome version: GRCh37 (hg19) | | | | | | | | | |
